# Perceived realism of haptic rendering methods for bimanual high force tasks: original and replication study

**DOI:** 10.1038/s41598-023-38201-x

**Published:** 2023-07-11

**Authors:** Mario Lorenz, Andrea Hoffmann, Maximilian Kaluschke, Taha Ziadeh, Nina Pillen, Magdalena Kusserow, Jérôme Perret, Sebastian Knopp, André Dettmann, Philipp Klimant, Gabriel Zachmann, Angelika C. Bullinger

**Affiliations:** 1 Professorship for Production Systems and Processes, Chemnitz University of Technology, Reichenhainer Straße 70, 09126 Chemnitz, Germany; 2 Department of Orthopedics, Trauma and Plastic Surgery, University Hospital Leipzig, Liebigstraße 20, 04103 Leipzig, Germany; 3 Division of Macroscopic and Clinical Anatomy, Gottfried Schatz Research Center, Medical University of Graz, Auenbruggerplatz 25, 8036 Graz, Austria; 4 Chair for Ergonomics and Innovation, Chemnitz University of Technology, Erfenschlager Straße 73, 09125 Chemnitz, Germany; 5 Chair of Computer Graphics and Virtual Reality, University of Bremen, Bibliothekstraße 5, 28359 Bremen, Germany; 6 Haption GmbH, Dennewartstraße 25, 52068 Aachen, Germany; 7 YOUSE GmbH, Florastraße 47, 13187 Berlin, Germany

**Keywords:** Mechanical engineering, Computational science, Orthopaedics

## Abstract

Realistic haptic feedback is a key for virtual reality applications in order to transition from solely procedural training to motor-skill training. Currently, haptic feedback is mostly used in low-force medical procedures in dentistry, laparoscopy, arthroscopy and alike. However, joint replacement procedures at hip, knee or shoulder, require the simulation of high-forces in order to enable motor-skill training. In this work a prototype of a haptic device capable of delivering double the force (35 N to 70 N) of state-of-the-art devices is used to examine the four most common haptic rendering methods (penalty-, impulse-, constraint-, rigid body-based haptic rendering) in three bimanual tasks (contact, rotation, uniaxial transition with increasing forces from 30 to 60 N) regarding their capabilities to provide a realistic haptic feedback. In order to provide baseline data, a worst-case scenario of a steel/steel interaction was chosen. The participants needed to compare a real steel/steel interaction with a simulated one. In order to substantiate our results, we replicated the study using the same study protocol and experimental setup at another laboratory. The results of the original study and the replication study deliver almost identical results. We found that certain investigated haptic rendering method are likely able to deliver a realistic sensation for bone-cartilage/steel contact but not for steel/steel contact. Whilst no clear best haptic rendering method emerged, penalty-based haptic rendering performed worst. For simulating high force bimanual tasks, we recommend a mixed implementation approach of using impulse-based haptic rendering for simulating contacts and combine it with constraint or rigid body-based haptic rendering for rotational and translational movements.

## Introduction

Whilst the visual rendering quality for Virtual Reality (VR) applications has dramatically increased in the last two decades, the quality of haptic feedback is severely lagging behind in simulation realism. This is especially crucial for VR applications for training motor-skills. Obliviously, procedural knowledge about the correct execution of a task is trainable with visuals/audio-only VR, but the most important, and often most difficult part, the motor-skill training, still has to be trained in reality since proper haptic simulations often do not exist or are not good enough.

A prominent application area where this can be observed is the training of surgical procedures using VR^1^. For procedures requiring the simulation of small forces, e.g. laparoscopic and arthroscopic procedures^2–6^, dentistry tasks^7,8^ or endoscopic surgeries^9^, there are suitable VR training simulators incorporating haptic feedback already available. Some residency curricula even require to train at such simulators^10,11^. In contrast, surgical procedures requiring the application of large forces, i.e. hip, knee and shoulder arthroplasty, lack VR training simulators with realistic haptic feedback. Here, solely visuals-only VR applications exist^12^, or they rely on pen-like haptic devices capable of providing only a fraction of the forces required for a realistic haptic simulation, e.g. over 300 N for Acetabula reaming during hips arthroplasty^13^. Devices like the Touch from 3D Systems, Inc., or the Omega series from Force Dimension are limited to forces up to 12 N^14,15^. Even the currently most advanced force feedback devices from 3D Systems, Inc., Force Dimension or Haption are currently limited to 20 N respectively to 35 N^16–18^. In order to overcome this shortcoming Sagardia et al.^19^, Kaluschke et al.^20,21^ or Knopp et al.^22^ were utilizing industrial robots like the KUKA LBR iiwa or its predecessors for providing force feedback up to 140 N (KUKA LBR iiwa 14).

Aside from the mechatronic capabilities of the haptic device, the utilized haptic rendering methods are crucial for the perceived haptic realism. A large body of work is available regarding these methods^23^; however due to the lack of high-force haptic devices these methods were never evaluated for forces above 35 N. In this study we aim to close this gap, by utilizing a Virtuose 6D prototype from Haption, capable of delivering 70 N force feedback. We focus on investigating the four most common haptic rendering methods:

1. Penalty-based haptic rendering (*penalty*)
2. Constraint-based haptic rendering (*constraint*)
3. Impulse-based haptic rendering (*impulse*)
4. Rigid-body-based haptic rendering (*rigid body*)

Each has different advantages and disadvantages and might perform relatively better than another rendering method only in a specific task. For example, *penalty* is advantageous if constraints should be contradictable, such as inserting a peg into a hole that is smaller than its diameter, but it cannot prevent interpenetration of objects. Constraint-based haptic rendering guarantees that constraints are not violated, and especially avoids pop-through issues of penalty, but is more complex to implement. The impulse-based method gives a sharper feeling of collision, but it cannot handle stable contact on its own. Finally, rigid-body-based rendering is closer to the real laws of physics, but it is much more computationally expensive. Those four rendering methods cover the vast majority of existing methods from the literature.

As forces of above 35 N are difficult to control one-handed, they are often performed two-handed. This, and the fact that two-handed interactions are rather less explored, are the reasons why we evaluated the four haptic rendering methods in a two-handed scenario.

**Figure 1:**
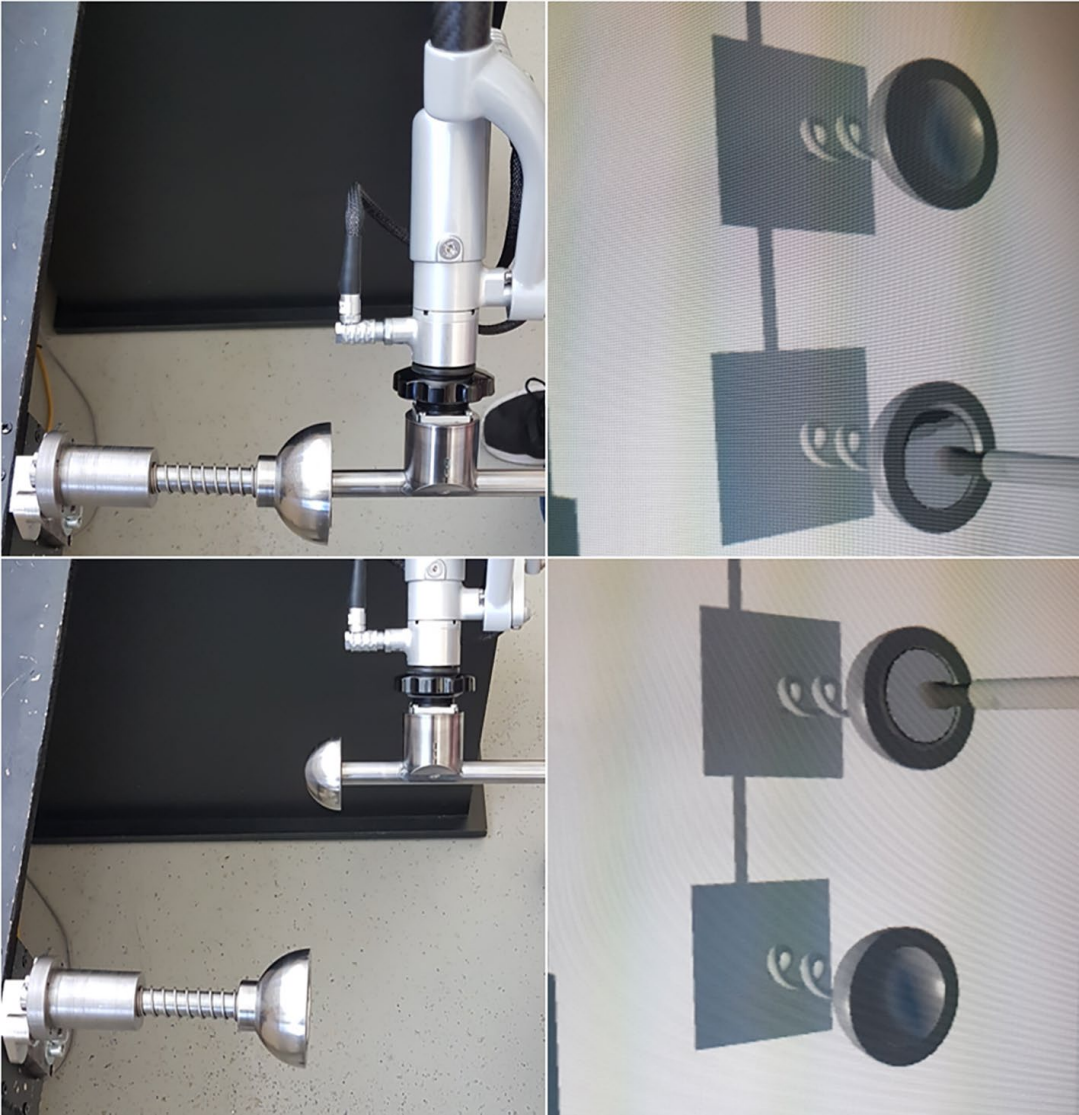
The tool in contact with the real haptic object (top left) and the corresponding virtual view in VR (top right); The tool in contact with the virtual haptic object (bottom left) and the corresponding virtual view in VR (bottom right).

**Figure 2:**
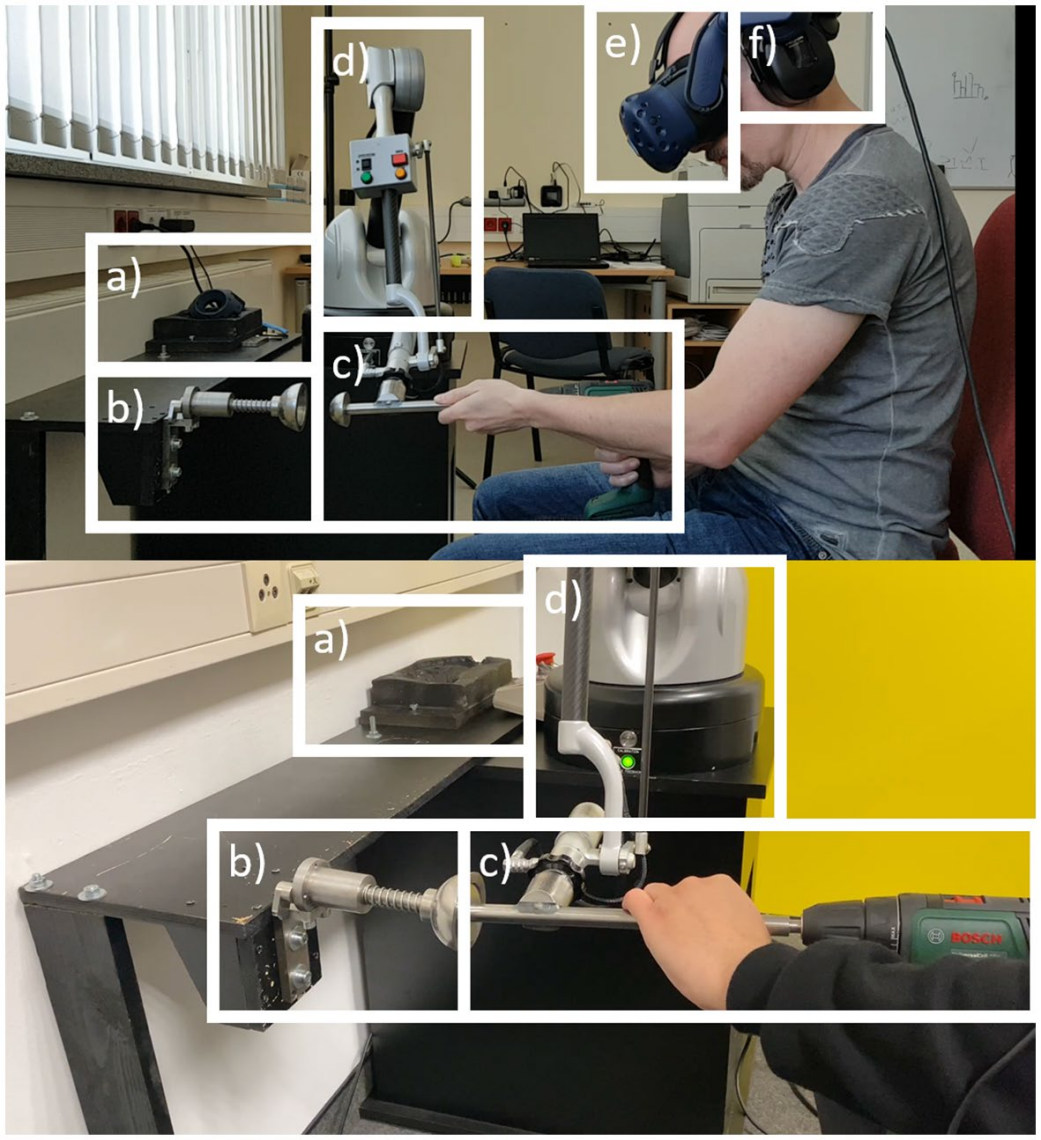
The experimental setup in the original study (top) and replication study (bottom). (**a**) Form for HTC VIVE controller (**b**) Real haptic object; (**c**) Hand-drill with attached rod and half-sphere; (**d**) Virtuose 6D haptic device; (**e**) HTC VIVE PRO; (**f**) Passive-noise cancelling headphones.

Our long-term goal is to provide realistic haptic feedback for hip arthroplasty where forces above 35 N are required for certain steps^13^. One example for this is the reaming of an Acetabulum (hip socket) during hip arthroplasty, which is done to prepare the Acetabulum geometry for the hip socket implant. The surgical tool used for this task resembles a hand-driller and is operated as such. To ream the Acetabulum, a rod with a half-spherical reamer is clamped by the tool holder of the surgical hand-driller. There is further one part around the rod which allows to grab it whilst rotating. Inspired by this task we designed and built an abstract evaluation scenario to compare the four haptic rendering methods in terms of perceived realism to a real scenario. By inserting a steel half-sphere attached at a rod, which is held by a hand-drill, into a steel cavity we can investigated the four haptic rendering methods (see Sects. “Experimental setup” and “Study design” as well as Figs. 1 and 2 for details).

The 2015 article by the Open Science Collaboration^24^ reported that the results of a large quantity of psychological studies published in high ranking journals could not be reproduced. This article fostered a debate about the so-called ‘replication crisis’, which also concerns the reproducibility of human subject research in Virtual, Augmented and Mixed Reality^25^. Unfortunately, replicating studies in Virtual, Augmented and Mixed Reality research is very rare, but highly needed to strengthen the trust in the reliability of the study results. This situation motivated us to conduct a replication study in addition to our original study using the same materials and experimental design but performed by a different research lab. Using this approach, we aim to strengthen the reliability of our results and would like to set a motivating example for other researchers in the field.

Our motivation to evaluate a steel/steel contact instead of a bone-cartilage/steel contact is based on three reasons. Firstly, bone and cartilage are biomaterials and as such their material properties vary largely between specimens unlike non-biomaterial like steel which material properties only have miniscule differences. Secondly, in a real surgical situation, the bone-cartilage part, e.g. the Acetabulum, is not rigid. It is surrounded by soft tissue and kinematically coupled via tendons and muscles to other bones, therefore, being a highly damping and moveable system. Here, the inter-specimen variety is even greater than the bone-cartilage material properties. Thoroughly evaluating haptic rendering methods in such a setting would lead to an unmanageable amount of studies to explore their performance. By abstracting this problem to a rigid steel/steel contact, we investigated the haptic rendering methods in a most challenging condition in terms of stiffness. We argue that our results present baseline data from which reliable estimations on the likely performance of the four investigated haptic rendering methods in high-force surgical scenarios can be drawn. As a third point, using a rigid steel/steel contact scenario allows us to provide conclusions for the simulation of other, non-surgical tasks, e.g. drilling into steel, concrete, wood, or screwing with an electrical screwdriver. 

The research questions we answer in this paper, in respect to the abstracted physical model used in the experimental tasks, are:

- RQ1. Is any investigated haptic rendering method capable of delivering realistic haptic feedback?
- RQ2. Which investigated haptic rendering methods delivers the highest degree of perceived realism across all tasks?
- RQ3. In which way do the investigated haptic rendering methods differ in perceived realism for different tasks?

### Contribution

Our study’s main contributions are:

1. Investigation of the four most common haptic rendering methods for forces above 35 N in terms of perceived realism.
2. Comparison of the four most common haptic rendering methods in a steel/steel contact scenario to reality.
3. Validation of the original study results by a replication study.

## State of the art

There have been numerous unique haptic rendering solutions presented in the last three decades. We do not intend to list each one of them. Rather, we will present four types of haptic rendering approaches which are most commonly employed throughout the literature, either rigidly or in various forms of hybrid combination. As such, this document provides a coarse overview of haptic rendering methods. For a more comprehensive list of approaches, we refer to recent literature reviews^23,26,27^.

Haptic rendering techniques can firstly be differentiated into two types of application methods, *direct* and *indirect* force rendering^28^. Direct force rendering renders forces which are exerted on a virtual object, which is simultaneously directly attached to the force rendering device. We will call this object the *haptic tool*^29^ if it is directly attached to the haptic device. In contrast, indirect force rendering methods employ a second instance of the same geometrical object, here called the *graphic tool*^29^, which position and rotation is calculated in a simulation loop and the difference between the poses of *graphic tool* and *haptic tool* are rendered to the haptic device, often as a dampened spring. The exact details of how the simulation is implemented varies greatly.

When looking at both categories of application methods, we can differentiate between four major haptic rendering techniques:

1. *Penalty* (Sect. “Penalty-based methods”)
2. *Constraint* (Sect. “Constraint-based methods”)
3. *Impulse* (Sect. “Impulse-based methods”)
4. *Rigid body* (Sect. “Rigid-body-based methods”)

Where *penalty* is commonly implemented as direct force rendering techniques, and *constraint, impulse* and *rigid body* are commonly implemented as indirect techniques.

The terms *penalty*-, *impulse*- and *constraint*-based methods also carry definitions in multi-body dynamics (MBD) simulations and should not be confused with the similarly named haptic rendering methods. In MBD, *penalty*-, *impulse*- and *constraint*-based describe methods that solve the dynamics of physical phenomenon, such as contacts, in a physically inspired manner. In that sense, they are variations of the *rigid body*-based method, from the view of haptic rendering. Here, the impulse-based method for example does not simulate rigid bodies, but essentially overlays a damping force during a collision, on top of the force that is rendered based on the tool’s configuration in space. In the following we will explain each method in more detail.

### Penalty-based methods

Penalty-based methods treat constraint violations of the tool, such as interpenetrations with the virtual environment, by measuring the amount of violation and applying a penalty force proportional to the violation. Less abstractly, if half of a sphere is overlapping with the environment, then a penalty force with a magnitude proportional to the overlapping will be applied to the sphere. The measure that is used to quantify the constraint violation can be implemented in different ways, most commonly is the depth of penetration (dop), either translation dop or generalized dop^30^, or volumetric measures^31,32^ or adaptive stiffness^28^. This rendering method is most commonly implemented as direct application. However, there are techniques to modify the method to be indirect application^33^, which can improve system stability. Penalty methods are easy to implement, as only discrete collisions and a penetration measure are needed. However, the disadvantages are plenty: constraints can be violated, such as overlap and discrete collision detection can miss a fast moving collision. The penetration measure may define the inside based on the current position, which can lead to pop-through events, when penetrating too far. Based on the fact that penalty violates real non-overlapping behavior, we expect this method to perform worse than all other methods in all tasks, especially when the normal force is high, such as during pushing.

### Constraint-based methods

Constraint-based rendering methods collect and solve non-penetration constraints that result from the virtual environment’s geometry contacts with the *graphic tool*, in order for it to remain on the surface without interpenetration. This interpenetration is of high importance in some industries, i.e. in construction in order to check if a car or plane can be assembled. Xu et al.^28^ showed clearly the benefit of constraint-based methods regarding overlap-free behavior. The computation time is usually proportional to the number of contacts and might dip significantly below the desired 1 kHz update-rate, as constraint solving is usually computation heavy. This is commonly mitigated by parallelizing the indirect rendering scheme and constraint solving^34^. However, some methods are even fast enough to allow for highly dynamic environments, such as streaming point clouds^35^ or material removal^36^. When the constraints are approximated very coarsely or linearized, these methods are commonly referred to as proxy-based methods. Constraint methods are usually the most complicated to implement, as the computational load needs to be processed at sufficiently fast speed in order to allow for interaction. Most time is spent calculating contact points and solving for the new movement of the tool under the current constraints. Usually, constraint methods exhibit very little intersection between tool and environment, such that it is not easily visible and in theory they could also guarantee intersection-free tool movement, if constraints are solved in parallel. Based on the fact that these methods are stable and don’t allow for visible overlap, we suspect they have overall good performance.

### Impulse-based methods

Constantinescu et al.^37^ introduced a combination of a penalty method with the addition of impulsive forces when a colliding impact is detected. That means the tool collides with a velocity magnitude that exceeds a certain threshold, as to not trigger on resting contact. The force impulses are calculated based on Newton’s restitution law, inspired by impulse-based rigid body simulation^38^. However, when it is employed in haptic rendering there is an increase in perceived hardness when coming into contact with virtual objects. Kuchenbecker et al.^39^ showed that adding event-driven transients significantly increases the perceived hardness of a constraint-based force computation. Similarly, to these transients, many short impulses arise when using impulse-based rendering, which suggests that these high-frequency responses are the reason for the increase in perceived hardness. The implementation can be done on top of any kind of underlying haptic rendering method, giving it a lot of flexibility. This method most likely performs especially well in tasks where collisions happen often, such as coming into contact.

### Rigid-body-based methods

A broad group of methods are built on top of a rigid-body simulation. Consequently, this opens up a vast and completely separate area of research. Popular commercial physics engines are PhysX^40^, Bullet^41^, and Havok^42^, as well as open source engines like ODE^43^ and Box2D^44^ for 2D cases. We refer to Bender et al.^45^ for a comprehensive and educational overview of rigid-body simulation approaches, as there are numerous ways to implement the underlying simulation. However, the integration with the haptic device is usually achieved in same way in all rigid-body simulation, with a dampened spring between *haptic* and *graphic tool*, which enables the haptic device to drive the simulation. The force that this spring exerts on the *haptic tool* is displayed to the user, as such it is a classic indirect application method. We suspect rigid-body-based methods will perform similarly well to constraint-based methods, since both are stable and don’t allow for visible overlap. However, hard contacts can likely not be rendered as well as with impulse-based methods.

## Methods

The four haptic rendering methods were investigated using a with-in-subject study design. Every participant compared the real object on the left with the virtual one on the right in every rendering condition and in order of three tasks. The study design for the original and the replication study were identical. The experimental setup is described in Sect. “Experimental setup”. The only differences were the location of the study and the principle investigator guiding the study. Sections “Implementation of haptic rendering methods” and “Study design” are providing the implementation of the haptic rendering methods respectively the study design. The statistical methods used are described in Sect. “Statistical evaluation” and the participants demographics in Sect. “Participants demographics”.

Ethical approval was obtained from Chemnitz University of Technology ethics committee (number: #101534678). All methods were performed in accordance with the relevant guidelines and regulations. All participants provided written and informed consent. A COVID-19 protection protocol, approved by the Chemnitz University of Technology and University of Bremen, was prepared and followed.

### Experimental setup

In order to ensure a similar body posture to acetabulum reaming, a seated setup was chosen where the participants should hold a hand-drill with an attached rod (steel, 15 mm diameter, 300 mm length) that had a half-sphere (steel, 50 mm diameter) at its end (see Fig. 2c). This hand-drill was connected to a novel Virtuose 6D prototype from Haption (see Fig. 2d). The prototype is an evolution of the standard Virtuose 6D, with higher torques on all motors, giving a maximum force of 70 N in translation (5 Nm in rotation) at the wrist in the whole workspace. The higher motor torques are achieved by larger reduction factors, so that the domain of stability increased and allows for a control stiffness up to 12 kN/m in translation (40 Nm/rad in rotation) with an update rate of 1 kHz. The connector to the Virtuose 6D was welded to the rod 60 mm behind the tip of the half-sphere. As an interaction counterpart a haptic object (see Fig. 2b) was chosen, with a cavity (steel, 50 mm inner diameter, 70 mm outer diameter) of the same size as the half-sphere at the hand-drill. This cavity was screwed to a steel rod (steel, 15 mm diameter, 128 mm length) which was running inside a bush (steel, 15 mm diameter, 60 mm length) allowing a guided uniaxial movement of the cavity. Between the cavity and the bush, a spring (spring constant = 1061 N/m) was mounted with a preload of 30 N, in order to enable a contact between both parts without the cavity moving. Further, 30 N is close to the upper boundaries of forces that commercially available haptic devices are able to deliver. Towards the end of the rod leaving the bush, a notch was milled. At the backside of the bush a lose lever was screwed, gliding on the rod until the notch appeared. The leaver would fall into the notch due to gravity therefore blocking the cavity in this position. By rising the lever, the cavity is released returning to the starting position. This mechanism allowed to push the cavity 27 mm in, staring from 30 to 60 N resisting force. This interval was chosen as it represents the upgraded force capabilities of the novel Virtuose 6D prototype. We decided to stay 10 N below its maximum capacity in order to avoid the participants over pushing the device. In order to attach this haptic object to a table a steel plate was screwed to the bush with two M8 drill holes.

The Virtuose 6D prototype with the attached hand-drill and the haptic object were mounted on a table in front of which the participants can be seated on a height adjustable chair. In order to block the auditory channel and any bias coming from here, the participants were wearing a passive-noise cancelling headset. Via this headset an additional white noise signal was conveyed to the participants (see Fig. 2f). The choice to use passive noise cancellation was due to the high frequency and transient nature of the noise, which is better blocked by passive techniques^46^.

A visual cue was essential to ensure that the participants hit the cavity with the hand-drill. Via an HTC VIVE PRO Head-mounted display (HMD) the participants saw a sparse virtual environment of the table with the hand-drill and two haptic objects next to each other, without a virtual body (see Figs. 1, 2e). The left haptic object was co-located with the position of the real haptic object. The right haptic object was placed 160 mm next to the left one and solely virtual (see Fig. 1). It was designed to deliver the haptic force feedback of the four haptic rendering methods. We ensured that the participants could not collide with the table whilst interacting with the right haptic object. The position of both haptic objects right next to each other was chosen carefully. This arrangement assures that the participant only had to move minimally to compare both haptic objects, while also keeping the kinematic chain of the Virtuose 6D almost unchanged. Thus, bias from the participants posture and the Virtuose’ kinematic chain was minimized. A visualization of this evaluation scenario was required so that the participants were able to make contact of the half-sphere with the cavity. This was implemented with the Unity 3D engine. In a blind-folded scenario, some sort of haptic guiding mechanism would have had to be designed, which would properly had led to a bias in the perceived haptics. The position of the hand-drill in VR was tracked via the Virtuose 6D. In order to have a smooth and stable visual movement when the cavity was pushed in, a collision of the half-sphere and the cavity was detected with Unity. The virtual movement of both cavities in VR was then done using the tracking information from the Virtuose 6D.

The alignment of the VR scenario with the real table and the haptic object was achieved with a form where an HTC VIVE controller could be placed (see Fig. 2a). This way the location and orientation of the HTC VIVE Controller in the VR frame was registered. The relative position of this form to the base of the Virtuose 6D was measured. The Virtuose 6D itself was screwed to the table at a fixed position. Based on this information the offset between the Virtuose 6D and the HTC VIVE Controller was computed, and the virtual camera in VR positioned accordingly. Due to tracking errors of the Lighthouse Tracking System a minimal, but still perceivable offset of the virtual and the real haptic object was sometimes present. Thus, the position of the virtual haptic object had to be slightly adjusted manually by the principle investigator in order to achieve a sufficient alignment.

Via a web-based interface the principal investigator switched the haptic rendering methods. Further, a Unity window enabled the principal investigator to follow the participants’ actions in VR and provided input fields for the initial alignment of the real and the virtual environment.

The entire software of the Virtuose 6D, the VR scene and the web-interface ran on a single PC with an Intel® Core™ i7-3770 CPU 3.40 GHz, 16.0 GB of RAM, a NVIDIA Quadro M6000 graphic card, and Windows 10.

### Implementation of haptic rendering methods

The following sections describe in detail the implementation of the four haptic rendering methods as used in the experiment. The choice of the methods reflects the taxonomy used in the review paper Zendejas et al.^4^.The forces and torques we describe in the following sections are calculated in relation to the virtual object, the hemisphere tool. However, before applying them to the haptic device, they need to be translated to the device in order for the contact to be perceived at the sphere’s location. The device force and torque are calculated in the following way:

$${F}_{device}=F,$$

$${\tau }_{device}=\tau +\left(\left({\mathrm{t}}_{\mathrm{h}}-{\mathrm{p}}_{\mathrm{device}}\right)\times \mathrm{F}\right),$$

where ${\mathrm{t}}_{\mathrm{h}}$ is the *haptic tool’s* sphere center, which coincides with the physical steel sphere mounted to the haptic device. ${\mathrm{p}}_{\mathrm{device}}$ is the control point of the haptic device. The Virtuose 6D device can display a maximum torque of 5 Nm at its control point, and the tool was designed in such a way that the offset to the center of the steel sphere does not exceed 7 cm in any direction. Therefore, forces up to 70 N can be rendered without saturating the torques of the haptic device.

All methods have been implemented from scratch, without the use of third-party libraries. Our implementation guarantees a very short execution time, and all methods achieve a stable update rate of 1 kHz.

Because the spring-damper parameters can significantly affect the perception of the haptic rendering, we made sure that their effective values were identical throughout all four haptic rendering methods. The limit of stability of the Virtuose 6D device at the sphere center was determined experimentally and set to k_l = 6000 N/m and b_l = 100 N/m/s. We used those values to simulate unyielding contact, i.e. for the end of travel of the rod. For the compression phase, we used k = 1481 N/m and b = 20 N/m/s, as these values best resemble the real spring. The spring preload of 30 N is simulated quite literally, i.e. the cavity does not move unless/before a force above 30 N is applied by the test subject."

#### Penalty-based methods

The first haptic rendering method uses penalization of the penetration distance. The virtual environment consists only of a single hemisphere cavity, which means we can assume outside is always pointing towards the center of the hemisphere. Now wherever the *haptic tool* is, we only need to determine if it is in contact with discrete collision detection. In case it is in contact we calculate the force from the *haptic tool’s* center ${t}_{h}$ towards the hemisphere’s center ${c}_{h}$ as a dampened spring: $F=k\left({c}_{h}-{t}_{h}\right)+b{v}_{h}$ with ${v}_{h}$ being the *haptic tool’s* velocity, *k* and *b* being the spring-damper parameters discussed above. Our method does not exhibit any pop-through.

#### Constraint-based methods

The second haptic rendering method uses a proxy constrained outside of contact^47^. As our scenario consists only of a single sphere (the geometry of the tool) constrained in a half-sphere cavity, our constraint is solved by translational projection of the *graphic tool* sphere ${t}_{g}$ towards the *haptic tool* ${t}_{h}$, with the obstacle being the cavity. This computation is very fast and geometrically exact, and therefore free of such artifacts as can be experienced with surface meshes or assemblies of geometric primitives. In our implementation, the reaction force is very similar to our penalty-based force, with the notable difference, that the tool does not visually penetrate the cavity. The force is then calculated by $F=k({t}_{g}-{t}_{h})+b{v}_{h}$.

#### Impulse-based methods

We combined the implementation of our constraint-based method (Sect. “Constraint-based methods”) with added impulsive forces according to Constantinescu et al.^37^ to represent impulse-based force rendering in our user study through a four channel teleoperation controller. The force rendering overall runs the same routine as the constraint-based method, except that there is an additional force overlay which comes into play when collisions occur. A collision is a new contact that has a non-zero, non-separating relative velocity. In that case the force overlay will display an impulse during this time window, based on the tool friction state, as well as relative normal and tangential velocity to the obstacle (for details see Constantinescu et al.^37^). In case of a sphere along a single movement axis, the formula 26 of the Constantinescu et al.^37^ simplifies to: ${F}_{env}=-\frac{(1+e)}{\Delta t}\dot{q}$ which is equivalent to an additional damping. In practice, we used the formula ${F}_{impulse}={b}_{impulse}{v}_{h}$ with ${b}_{impulse}=300 \mathrm{N}/\mathrm{m}/\mathrm{s}$ which acts as an additional force overlay to the force calculation given in Sect. “Constraint-based methods”.

#### Rigid-body-based methods

In the last haptic rendering method, the proxy is managed by a rigid-body simulation using the approach of Ortega et al.^34^. The *graphic tool’s* trajectory is interactively simulated based on the *haptic tool’s* position ${t}_{h}$ and *graphic tool’s* position ${t}_{g}$. A difference between them results in an acceleration of the *graphic tool* $a=\frac{k}{m}{(t}_{h}-{t}_{g})$ with $m=9.87 kg$ (mass of the real tool). We project the acceleration, such that it does not violate the non-penetration constraint. The acceleration is then integrated to yield velocity (which is projected onto the constraint as well) and thus updates the position of the *graphic tool*. The force display is calculated similar to the other methods $F=k({t}_{g}-{t}_{h})+b{v}_{h}$ .

### Study design

**Figure 3:**
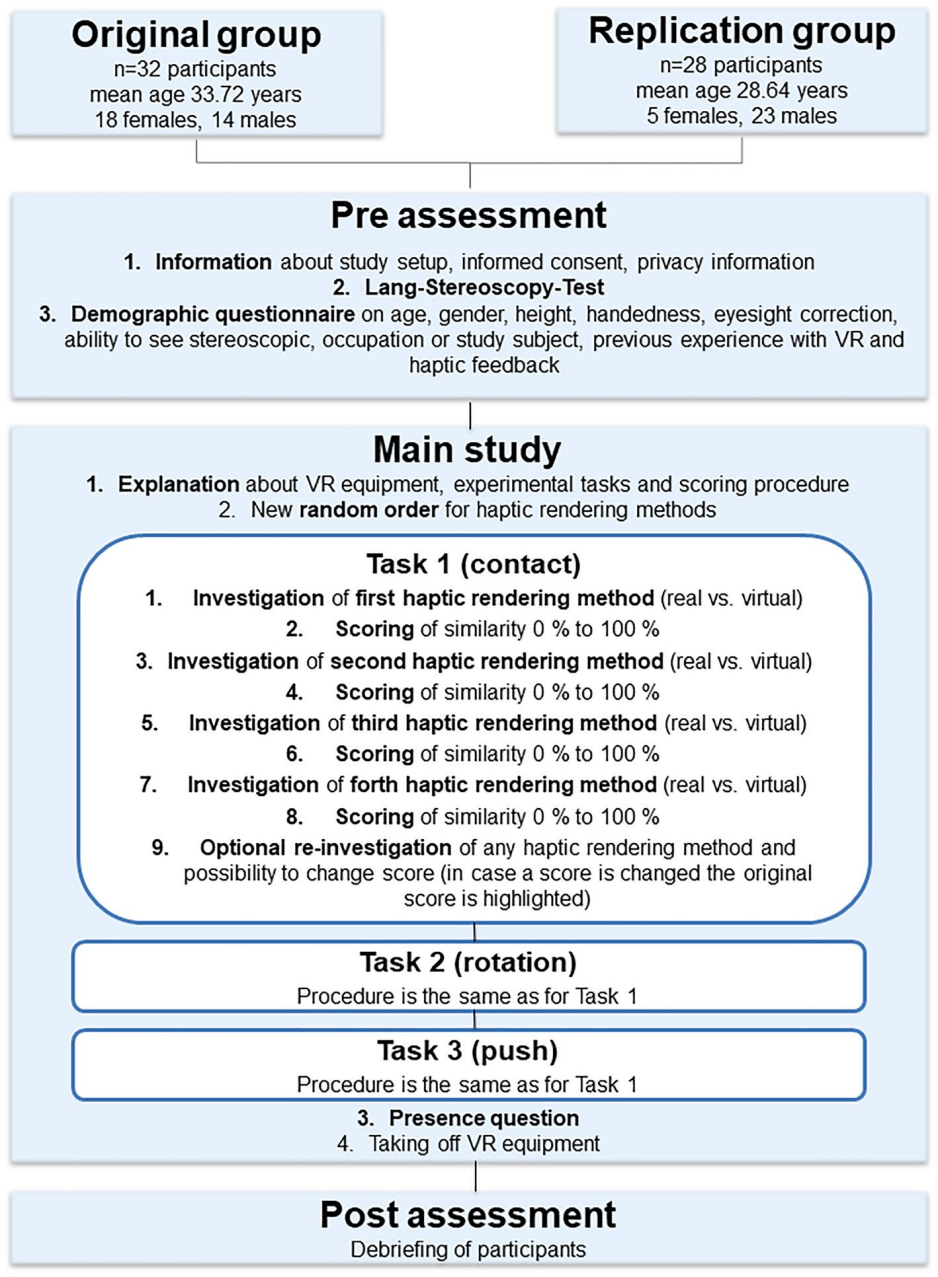
Graphical overview of the most important steps of the study protocol.

A written study protocol including all instructions given to the participants from welcoming them to their debriefing was prepared and followed during the experiment. It consisted of three parts: (1) pre-assessment, (2) main study and (3) post-assessment (see Fig. 3).

After welcoming the participants, they were informed about the study setup and data privacy verbally and written. After giving their written informed consent the participants filled out the demographic’s questionnaire providing information about age, gender, height, handedness, eyesight correction, ability to see stereoscopic, occupation or study subject, previous experience with VR and haptic feedback. The stereoscopic vision of each participant was additionally checked by the principal investigator using the Lang-Stereoscopy-Test^48^ Lang Stereotest II from LANG-STEREOTEST AG, Switzerland.

The main-study-part began with the participants adjusting the height of the chair where they sat in front of the experimental setup (see Fig. 2). The principal investigator explained the VR and haptic devices as well as the experimental tasks and how the participants should score the realism of the haptic rendering methods. Next, they were presented with three consecutive tasks. In each tasks the participants investigated all four haptic rendering methods which order was randomized between participants. We made sure to uniformly sample the permutations of the possible order of methods to minimize the effect of the order on the results. The order of tasks was content-related and could not be randomized.

**Figure 4:**
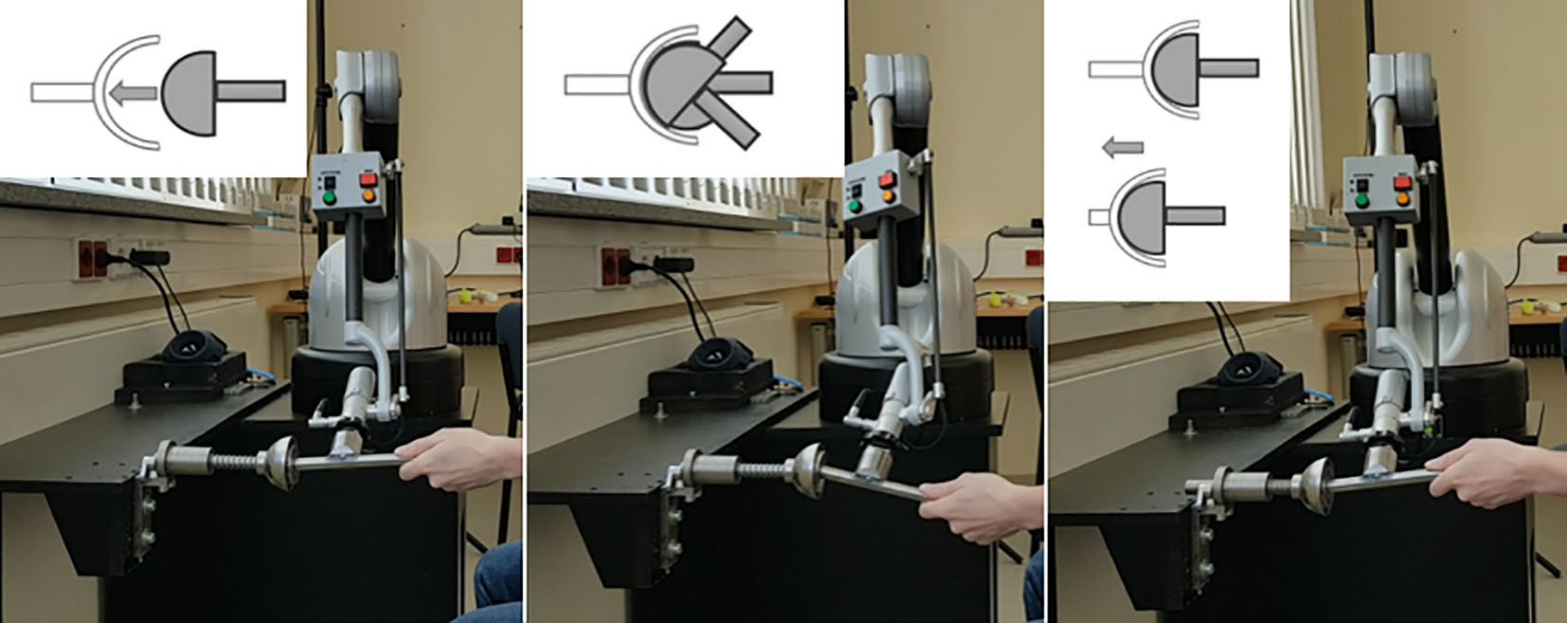
Task 1: Making contact between the tool and the haptic object (left); Task 2: Rotating the tool in the cavity of the haptic object (center); Task 3: Pushing-in the haptic object with the tool (right).

In Task 1 the participants should only make contact between the half-sphere attached at the hand drill and the cavity, which corresponds to placing the reamer on the acetabulum during real surgery (see Fig. 4, left). Task 2 consisted of rotating the hand-drill inside the cavity, which corresponds to finding the correct angle for reaming during real surgery (see Fig. 4, center). Here the participants were instructed to only pay attention to the feeling of rotation and not to push the hand-drill. In Task 3 the participants were asked to push the haptic object with the hand-drill until it either blocked (real) or was highlighted green (virtual; see Fig. 4, right). They were instructed only to include the sensation of uniaxial movement into their score but not the blocking/highlighting. This corresponds to the actual reaming during real surgery when material is removed. Inspired by Park et al.^49^ each haptic rendering method was scored on a scale from 0 to 100%. Therefore, the participants were always asked “Please score now how close the simulation on the right side resembles the real experience on the left side. Please provide a value between 0 and 100%”. This question was adapted from literature investigating haptic feedback^39,50–54^.

Then the participants put on a passive noise cancelling headphones and the HMD. After the participants were familiar with the virtual environment, the hand drill was given to them and Task 1 was explained again. After the participants said that they were ready, white noise was given via the headphones and the first haptic rendering method was presented. After they stopped investigating the haptic object with the hand drill the principal investigator turned off the white noise, asked for their scoring and noted it. The second rendering method and the white noise were activated so that the participants could investigate the second haptic rendering method. This procedure continued until all four haptic rendering methods in all three tasks were investigated and scored by the participants. The participants were able to retry any rendering method during a task and could also change their scoring. The principal investigator reminded them of these options at the end of each task. If the participants changed a score, the first score was noted and highlighted. After the completion of all three tasks the hand drill was taken from the participants but they remained seated, still wearing the HMD. The principal investigator asked the participants for an overall ranking of all for haptic rendering methods from the best to the worst. This question was not disclosed to the participants earlier in order to not influence their subjective rating. Therefore, the principal investigator made sure that the participants always knew if they were currently interacting with the first, second, third or fourth during the investigation of the haptic rendering methods in each task. Next, the participants were asked to rate their subjective presence on a 1 to 10 scale inside the virtual environment using Bouchard et al. single-item measure “To which extend do you feel present m the virtual environment, as if you were really there”^55^ following the advice of Skarbez et al.^56^ for the application of presence questionnaires. The presence measure was used to control the influence of visual cues in the evaluation of perceived realism, assuming that sufficient presence means no or only little irritations via the VR technology are given. Lastly, the participants took off the HMD and headphones.

During post-assessment the participants were asked about their well-being and any questions regarding the experiment were answered by the principal investigator.

The participants of the study were recruited using mailing lists of the Chemnitz University of Technology and University of Bremen and social media. Only participants aged over 18, with stereoscopic vison and normal or corrected eyesight (contact lenses or glasses) were included.

### Statistical evaluation

Data analysis was carried out with IBM SPSS Statistics 28. To compare the demographics of the original and the replication study samples, *t* tests for independent samples were calculated.

To identify differences of the perceived realism between the haptic rendering methods and between the tasks, a two-way repeated measures ANOVA was performed. The according preconditions were checked using a Shapiro–Wilk-test to examine the residues for normal distribution, followed by analyzing a boxplot for outliers. Mauchly’s test of sphericity was calculated to check the variances of differences between all possible pairs of conditions. If sphericity was not fulfilled Greenhouse–Geisser correction was applied. Pair-wise *t*-tests with Bonferroni correction were used as post-hoc tests.

Differences between original and replication study in relation to the rendering conditions and tasks were analyzed with a mixed ANOVA. *P*-values ≤ 0.05 were considered as statistically significant. Partial eta squared (η^2^_p_) and Cohen’s *d*_z_ as effect sizes were interpreted according to conventions of Cohen^57^.

To verify the consistency of the overall ranking of the four haptic rendering methods and their perceived realism in the tasks Spearman’s rho (ρ) as rank correlation coefficient was calculated. For this the perceived realism assessment of every rendering method was summarized over all tasks.

### Participants demographics

The original study included 32 subjects – 18 females and 14 males (self-identified). Individuals age ranged from 22 to 57 years (*M* = 33.72, *SD* = 9.25). Two of the subjects were left-handed, 22 subjects wore glasses or contact lenses during the study. All participants in the original study passed the Lang-Stereoscopy-Test, ensuring their ability to correctly perceive stereoscopic images. For the replication study 29 participants were recruited. One person failed the Lang-Stereoscopy-Test and was excluded from the study. The remaining 28 subjects age ranged from 20 to 55 years (*M* = 28.64, *SD* = 7.99). Of those, 5 self-identified as female and 23 as male. One of the subjects was left-handed, 16 subjects were wearing glasses or contact lenses during the study (see Table 1). In both studies most of the participants were experienced with VR and some with haptic feedback. The two study samples differ significantly in age and body height. The original study sample is older (*t*(58) = 2.26, *p* = 0.028) and smaller (*t*(58) = 2.48, *p* = 0.016).

**Table 1:** Demographics of original and replication study.

Variable	Value	Original study N = 32	Replication study N = 28
Gender	Female	18 (56.3%)	5 (17.9%)
	Male	14 (43.8%)	23 (82.1%)
	Diverse	0	0
Age [years]		*M* = 33.72	*M* = 28.64
		*SD* = 9.25	*SD* = 7.99
		Range = 22–57	Range = 20–55
Body height [cm]		*M* = 173.13	*M* = 178.79
		*SD* = 9.30	*SD* = 8.28
		Range = 159–193	Range = 164–196
Handedness	Right	30 (93.8%)	27 (96.4%)
	Left	2 (6.3%)	1 (3.6%)
Visual aid	No visual aid	10 (31.3%)	12 (42.9%)
	Glasses	14 (43.8%)	12 (42.9%)
	Contact lenses	4 (12.5%)	3 (10.7%)
	Glasses & lenses	4 (12.5%)	1 (3.6%)
Occupation	Researchers	18 (56.3%)	8 (28.6%)
	Student	6 (18.8%)	18 (64.3%)
	Other	8 (25.0%)	2 (7.1%)
VR experience	Yes	24 (75.0%)	27 (96.4%)
	No	8 (25.0%)	1 (3.6%)
Haptic experience	Yes	13 (40.6%)	15 (53.6%)
	No	19 (59.4%)	13 (46.4%)

## Results

Section “Performance of haptic rendering methods in original study” provides the results of the perceived realism of the haptic rendering methods for the original study. In Sect. “Performance of haptic rendering methods in replication study” the results of the replication study are described. In Sect. “Performance of haptic rendering methods: original study vs. replication study” the two studies are compared. Section “Subjective feedback” includes selection of comments from the participants and observation from the principle investigators from both studies. Section “Summary” is briefly summarizing all results.

### Performance of haptic rendering methods in original study

**Figure 5:**
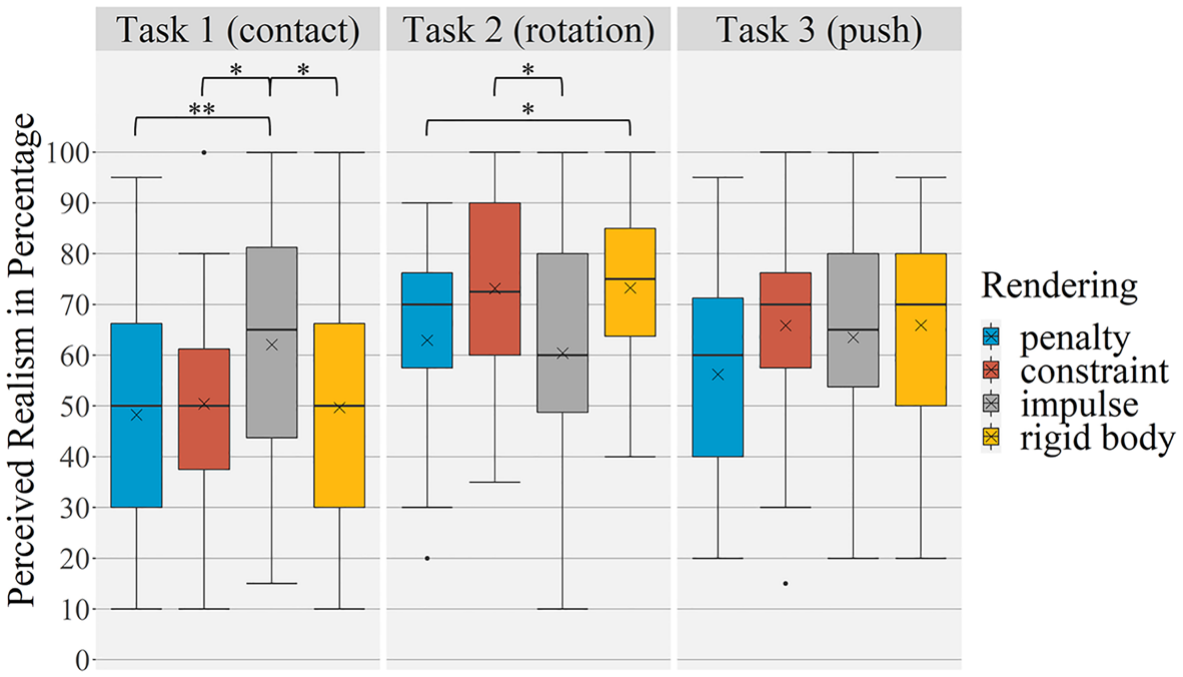
Boxplots of perceived realism of haptic rendering methods in the original study. x indicates the mean value, * indicates a significant difference of *p* < 0.05, ** indicates a significant difference of *p* < 0.01.

Mean perceived realism values of all haptic rendering methods vary between 48.28 (*SD* = 23.78) and 73.28 (*SD* = 15.17; see Table 2). Before calculating the ANOVA, the assumptions were checked and confirmed. The two-way ANOVA shows a significant main effect for differences between the haptic rendering methods (Greenhouse–Geisser *F *(2.13; 65.90) = 4.02, *p* = 0.021, η^2^_p _= 0.12). The post-hoc tests for all haptic rendering methods independent of the task are significant for differences between *penalty* and *constraint* (*p* = 0.006; *d*_*z* _= – 0.65) and between *penalty* and *rigid body* (p = 0.018; *d*_*z*_ = 0.57). *Penalty* was perceived less real than all other haptic rendering methods (see Table 2 and Fig. 5). The main effect for the task is also significant and reveals the highest effect size (Greenhouse–Geisser *F* (1.42; 44.13) = 9.14, *p* = 0.002, η^2^_p _= 0.23). The post-hoc tests show that Task 1 has a significant lower mean than Task 2 (*p* = 0.001; *d*_*z* _= − 0.70; see Table 2). Further, the interaction between haptic rendering method and task is significant (*F *(6, 186) = 5.83, *p* < 0.001, η^2^_p _= 0.16). The comparison of the haptic rendering methods in relation to the different tasks shows significant results for Task 1 and Task 2. In Task 1 the perceived realism evaluation for *impulse* was significantly higher than for *penalty* (*p* = 0.001;* d*_*z* _= 0.73), *constraint* (*p* = 0.016; *d*_*z* _= 0.58) and *rigid body* (*p* = 0.017; *d*_*z*_ = 0.58). In Task 2 the post-hoc tests shows significant differences between *penalty* and *rigid body* (*p* = 0.024; *d*_*z* _= − 0.55) as well as between *constraint* and *impulse* (*p* = 0.023; *d*_*z* _= 0.55).

**Table 2:** Perceived realism of haptic rendering methods in the original study for each task.

Task	Rendering	M	SD
1 (contact)	Penalty	48.28	23.78
	Constraint	50.43	20.64
	Impulse	62.09	23.88
	Rigid body	49.68	21.99
2 (rotation)	Penalty	62.97	19.46
	Constraint	73.12	17.76
	Impulse	60.43	22.44
	Rigid body	73.28	15.17
3 (push)	Penalty	56.25	19.05
	Constraint	65.86	18.37
	Impulse	63.53	20.43
	Rigid body	65.91	19.96

The frequencies of ranking order were similar distributed. Only *penalty* was evaluated mostly as worst (17 of 32). The ranking results for the other haptic rendering methods revealed an inconclusive picture with no clear results. There, was a correlation between perceived realism of *impulse* summarized over all tasks and its rank (ρ = − 0.51, *p* = 0.003). All other rankings indicate no correlation with the perceived realism of rendering methods.

**Figure 6:**
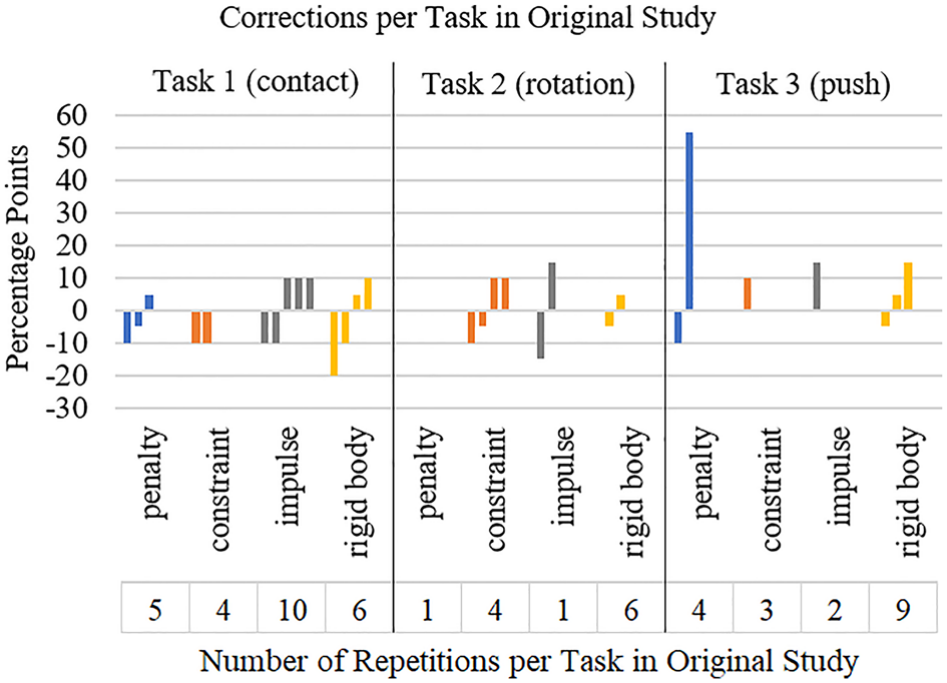
Correction values and number of repetitions for each haptic rendering method for each task in the original study.

Presence was rated with a mean of 7.65 (*SD* = 1.52). In total the participants repeated a haptic rendering method in 55 of 384 cases and corrected it in 29 cases. Corrections ranged from 5 to 20 percentage points with a single exception of 55 percentage points. In 33 cases the testing was repeated without correction. Nine times the testing was corrected without repetition. Task 1 was repeated (25 times) and corrected most often (14 times). Out of the haptic rendering methods *rigid body* with 21 repetitions and 9 corrections was the most repeated and corrected one (see Fig. 6).

### Performance of haptic rendering methods in replication study

**Figure 7:**
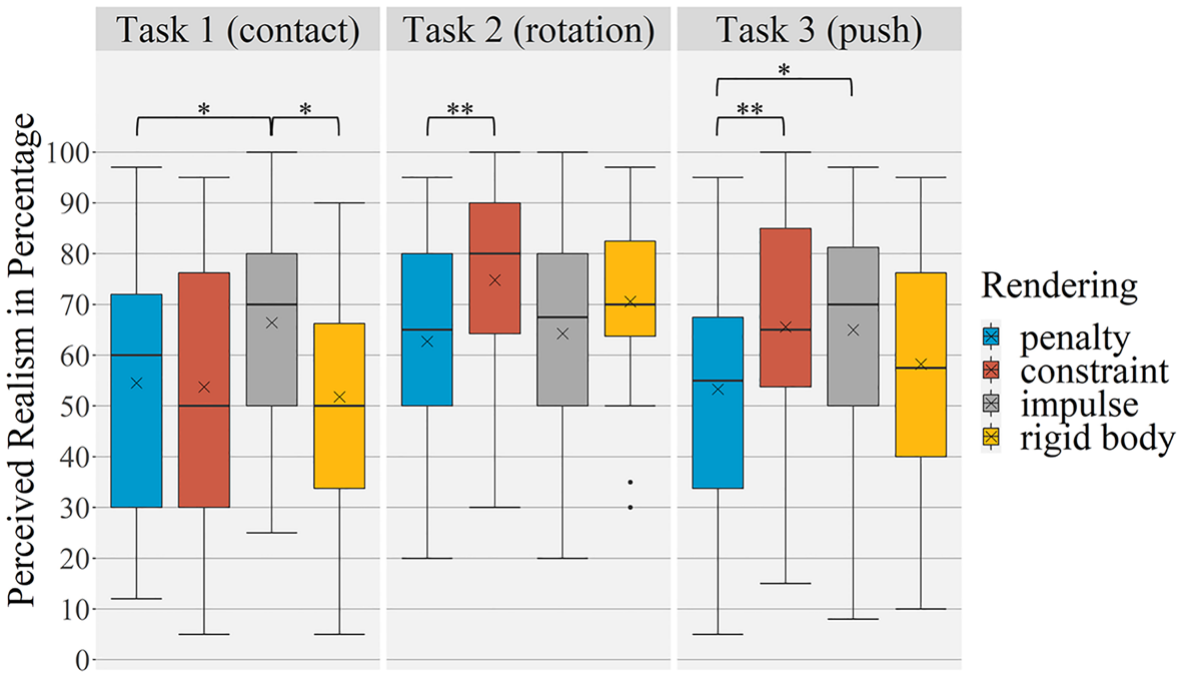
Boxplots of perceived realism of haptic rendering methods in the replication study. x indicates the mean value, * indicates a significant difference of *p* < 0.05, ** indicates a significant difference of *p* < 0.01.

Mean perceived realism values of all rendering methods vary between 51.82 (*SD* = 22.89) and 74.82 (*SD* = 16.60) (see Table 3). The assumption for ANOVA were also checked and confirmed. The two-way ANOVA shows a significant main effect for the differences between the haptic rendering methods (*F* (3; 81) = 4.41, *p* = 0.006, η^2^_p_ = 0.14). The post-hoc tests for all haptic rendering methods independent of the task are significant for the differences between *penalty* and *constraint* (*p* = 0.007; *d*_*z*_ = − 0.64) and between *penalty* and *impulse* (*p* = 0.030; *d*_*z*_ = − 0.56). *Penalty* was perceived less realistic than all other rendering methods. The main effect for Task is also significant and shows the highest effect size (*F* (2; 54) = 9.60,* p* < 0.001, η^2^_p_ = 0.26). In the post-hoc tests significant differences between Task 1 and Task 2 (*p* < 0.001; *d*_*z*_ = − 0.96) and also between Task 2 and Task 3 (*p* = 0.013; *d*_*z*_ = 0.61) are evident. Task 2 shows the highest mean value of perceived realism of all tasks (see Table 3). Further, the interaction between haptic rendering method and task is significant (Greenhouse–Geisser *F* (4.41; 118.97) = 5.02, *p* < 0.001, η^2^_p_ = 0.157). The comparison of the haptic rendering methods in relation to the different tasks show significant results for all tasks. In Task 1 the perceived realism for *impulse* was significantly higher than for *penalty* (*p* = 0.029; *d*_*z*_ = 0.58) and for *rigid body* (*p* = 0.016; *d*_*z*_ = 0.59). In Task 2 the post-hoc tests reveal that *constraint* is rated significantly higher than *penalty* (*p* = 0.001; *d*_*z*_ = 0.79). In Task 3 *penalty* was perceived as significantly less realistic than *constraint* (*p* = 0.006; *d*_*z*_ = − 0.68) and *impulse* (*p* = 0.017; *d*_*z*_ = − 0.59; see Table 3 or Fig. 7).

**Table 3:** Perceived realism of haptic rendering methods in the replication study for each task.

Task	Rendering	M	SD
1 (contact)	Penalty	54.54	24.62
	Constraint	53.75	26.63
	Impulse	66.43	19.95
	Rigid body	51.82	22.89
2 (rotation)	Penalty	62.75	21.86
	Constraint	74.82	16.60
	Impulse	64.29	22.42
	Rigid body	70.57	16.98
3 (push)	Penalty	53.32	23.77
	Constraint	65.57	24.13
	Impulse	65.00	23.18
	Rigid body	58.25	23.87

The frequencies of ranking order were similar distributed. Only *penalty* was evaluated mostly as worst (11 of 24). The ranking results for the other haptic rendering methods revealed an inconclusive picture with no clear results. There was a correlation between perceived realism of *rigid body* summarized over all tasks and its rank (ρ = − 0.631, *p* = 0.001). All other rankings indicate no correlation with the perceived realism of rendering methods. Three times no ranking was given.

**Figure 8:**
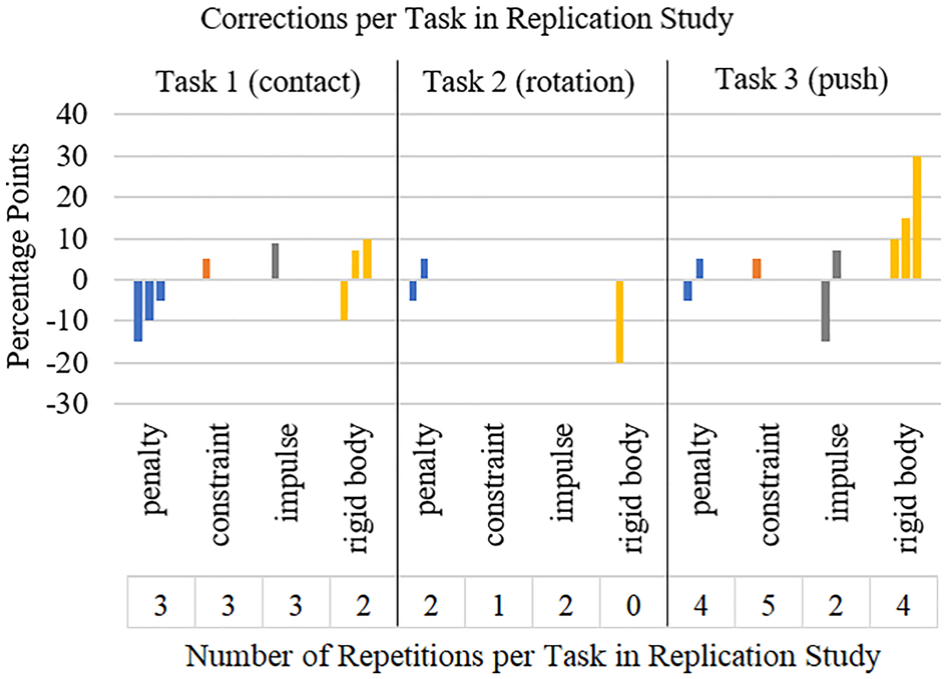
Correction values and number of repetitions for each haptic rendering method for each task in the replication study.

Presence was rated with a mean of 7.50 (*SD* = 1.30). In total the participants repeated a haptic rendering method in 31 of 336 cases and corrected it 20 times. Corrections ranged from 5 to 30 percentage points (see Fig. 8). In 13 cases the testing was repeated without correction. Three times the testing was corrected without repeating. Task 3 was repeated (15 times) and corrected most often (9 times), *penalty* and *constraint* were the most often repeated haptic rendering methods (9 times), *penalty* and *rigid body* were corrected most often (7 times). In the replication study there are also negative correlations of participants height and perceived realism of the haptic rendering methods, showing that taller participants scored lower on the perceived realism. This was found in Task 1 for *penalty* (*r* = − 0.514, *p* = 0.006) and *constraint* (*r* = − 0.55, *p* = 0.002), in Task 2 for *penalty* (*r* = − 0.54, *p* = 0.003) and *impulse* (*r* = − 0.54, *p* = 0.003) and in Task 3 for *penalty* (*r* = − 0.47, *p* = 0.013) and *rigid body* (*r* = − 0.41, *p* = 0.029).

### Performance of haptic rendering methods: original study vs. replication study

**Figure 9:**
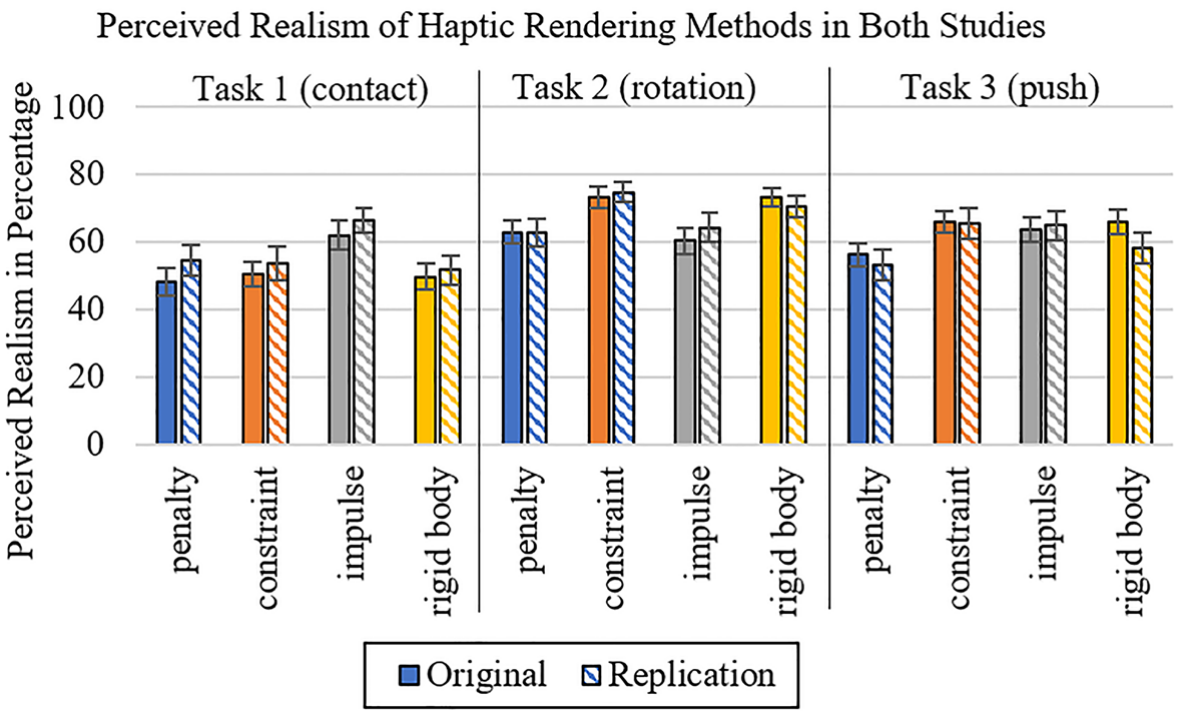
Comparison of perceived realism of haptic rendering methods in both studies (error bars = standard error).

The distribution of means of the descriptive values is very similar for both studies (see Fig. 9). The mixed ANOVA shows no significant effect for the between-subject factor (*F* (1; 58) = 0.05, *p* = 0.829, η^2^_p_ = 0.00), meaning that there is no significant difference between the original and the replication study at all. The examination of the within-subject factors reveals a significant effect for haptic rendering method (Greenhouse–Geisser *F* (2.43; 140.71) = 7.51, *p* < 0.001, η^2^_p_ = 0.12) and task (Greenhouse–Geisser *F* (1.55; 89.96) = 16.79, *p* < 0.001, η^2^_p_ = 0.22). Further, the interaction of haptic rendering method and task is significant (*F* (6; 348) = 10.46, *p* < 0.001, η^2^_p_ = 0.15). The interactions between haptic rendering method and the two studies (Greenhouse–Geisser *F* (2.43; 140.71) = 0.97, *p* = 0.395, η^2^_p_ = 0.02), between task and the two studies (Greenhouse–Geisser *F* (1.55; 89.96) = 0.98, *p* = 0.360, η^2^_p_ = 0.02) and also the interaction between haptic rendering method, task and the two studies together (*F* (6; 348) = 0.31, *p* = 0.934, η^2^_p_ = 0.01) are not significant.

### Subjective feedback

Throughout the study a lot of comments from the subjects were collected to find out what they are thinking about the perceived realism of the haptic rendering method and the study in general. The principal investigator of the original study noticed that about eight subjects operated very careful and slow with the hand-drill. Two other subjects (original study) moved the hand-drill in a rugged way. Eight subjects rotated the tool in huge radiuses (7 in original, 1 in replication study).

The comments about the perceived realism were ordered by task and haptic rendering method to see if participants tended to comment more on the tasks or the haptic rendering methods. Similar comments accumulated for tasks rather than haptic rendering method. In Task 1 of the original study, all rendering methods were described like too soft (“Feels soft”, “much softer than in reality”). Only one person in the original study commentated for *impulse*: “feeling that edges are really there”. The rotation performance of haptic rendering methods in Task 2 was partially characterized as “floating”, “swinging” or “elastic” and also “too soft” (original study). The evaluation of *impulse* in Task 2 was described with “scraping a bit” (2 in original study), “feeling like sand in between” (1 in original study) or “the virtual is like vibrating” (3 in original, 1 in replication study). In Task 3 four subjects mentioned for *penalty*, *constraint* and *rigid body* that the end of the haptic object was slipping through the cavity (original study).

Across all tasks two subjects in the original study commented that “all in all a hard stop is missing”. One person in the replication study said at the end of the study: “[*constraint*] and [*impulse*] should be combined, [*impulse*] for getting in contact, [*constraint*] for movement on the surface”. Another subject in the original study said: “[*impulse*] was the best in force but felt very rough as if splinter was inside. [*penalty*] and [*constraint*] felt like rubber”.

In both studies multiple participants expressed their difficulties in forming an overall ranking as they could not make out a clear winner and stated this explicitly. In the replication study this insecurity resulted in the inability of three participants to provide an overall ranking.

### Summary

In both studies’ significant differences of perceived realism in relation to the haptic rendering method and task could be found. Further, the pairwise comparisons of the haptic rendering methods in detail show similar results in both studies. The ranking of rendering methods did not correlate with the perceived realism of the rendering methods in the tasks.

## Discussion

The original and replication study are discussed in Sect. “Original study vs. replication study” before common limitations are given in Sect. “Limitations”.

### Original study vs. replication study

The results for the haptic rendering methods in the original and the replication study are very similar with only miniscule differences. The mean values of perceived realism of all haptic rendering methods are located in the mid-upper area of the scale, with mostly slightly higher values in the replication study. Further, the perceived realism differs in relation to the four haptic rendering methods and the three tasks in both studies, which is underlined by the significant interaction effects between haptic rendering method and task in both studies. In both studies the tasks were rated significantly different with Task 2 emerging as the highest rated, meaning that rotation could be simulated best.

*RQ1 “Is any investigated haptic rendering method capable of delivering a realistic haptic feedback?”.* Although all haptic rendering methods performed fairly well in the original and replication study, there is no haptic rendering method which could be considered delivering an indistinguishable haptic feedback from the interaction with the real haptic object for the given tasks. This is not surprising given the worst-case scenario of a steel/steel contact. Since it is well known that haptic systems become less stable if stiffness is increased^58^ and steel is one of the stiffest materials. However, we would assume that in a less stiff scenario a realistic sensation is achievable. Considering the acetabula reaming scenario where a bone-cartilage/steel contact with additional damping from the surrounding soft tissue is present, a realistic haptic feedback seems achievable.

*RQ2 “Which investigated haptic rendering methods delivers the highest degree of perceived realism across all tasks?”.* The results of both studies show that there is no clear best haptic rendering method as neither haptic rendering method performed best in all three tasks. This is further substantiated by the participants’ difficulties of forming an overall ranking. However, *penalty* clearly emerged as the worst from both studies, which was to be expected. This method’s known stability problems and the reduced realism due to visual artefacts, such as overlapping, clearly led to its worst performance. In addition, the results of the ranking, in which *penalty* was most frequently ranked as worst, confirms the quantitative findings. Though, it clearly emerges, that *constraint* and *rigid body* were rated very similar in every task in both studies. This means that the *rigid body* simulation did not have any benefit over direct geometric projection to solve constraints on the perceived realism.

*RQ3 “In which way do the investigated haptic rendering methods differ in perceived realism for different tasks?”.* Both studies showed that the haptic rendering methods were rated differently for each task. For Task 1 both studies show that *impulse* performed better than the other three haptic rendering methods which scores barely differed. That the steel/steel contact was simulated best by *impulse* confirmed our expectations from literature. The results for Task 2 show a clear grouping for *penalty* and *impulse* in a low rated group and *constraint* and *rigid body* in a high rated group. The fact that *penalty* was rated low was expected, as the visual artefacts are permanently shown in this task. It is interesting, that *impulse* performed worse, as this behavior was not documented before. We suspect that the small impulses that result from momentarily losing and coming into contact did not feel realistic. For Task 3 *penalty* emerged as the worst method from both studies whilst *impulse* and *constraint* performed similarly good. A difference could be found for *rigid body*, which had almost equal results than *constraint* and *impulse* in the original study, but was reported in-between *penalty* and the top group in the replication study. Although, this particular difference of *rigid body* to the top group was not significant, we would recommend focusing on *impulse* and *constraint* when selecting a haptic rendering method for applications resembling Task 3.

The presence ratings were almost identical in both studies and showed that a sufficient presence of the participants in VR could be achieved, despite the minimalistic design of the virtual environment.

The analysis of the overall ranking question showed in both studies that the participants had difficulties to give an answer which is evident from their comments and the found correlations. Nonetheless, this backs the quantitative results that there is no best haptic rendering method. Further, the ranking also confirmed that *penalty* gave the worst haptic feedback.

In terms of repetitions there were less in the replication study but with a higher correction rate than in the original study. Though, both studies showed similar moderate correction values which resulted in the same conclusion, that the option for repeating and correcting the rating might not have been necessary.

The most surprising difference between the original and the replication study was the negative correlation of the participants height on the perceived realism of the haptic rendering methods in the replication study which was not present in the original study. The reasons therefore are highly speculative but might lay in the fixed height of the table respectively the arm rests of the chair used for the replications study, as the one used in the original study had none. Aside from this, the samples did not differ in any other demographic variable except age which, however, did not had an influence on the results.

Lastly, the comments from the participants and the observations from the principal investigators in both studies were similar with no particular difference. This further substantiates the conclusions drawn from both studies.

Summarizing, we can state that no haptic rendering method was able to deliver a sensation of a real steel/steel interaction, although a realistic behavior in less demanding scenarios seems likable. In any case we would recommend not to use *penalty*. Instead, implementations should concentrate on combining *impulse* with *constraint* or *rigid body* to achieve a realistic hard contact behavior in conjunction with a smooth rotation and force built up. Though, the detection of the transition point and the assurance of a consistent haptic behavior will be a challenging feat.

### Limitations

The four haptic rendering methods were only investigated on one haptic device, the Virtuose 6D. The findings of this study should therefore be investigated with other haptic devices. Currently, only robots like the KUKA LBR iiwa are capable of delivering similar high forces. Our findings are based on three simple tasks. Future studies should incorporate more complex movements. The order of the tasks in this experiment was not randomized to reflect the sequence during acetabula reaming. Further, the measure to give an overall ranking at the end of the experiment requested prevented a randomization of haptic rendering methods between tasks. However, we could not detect a bias of data. The position of the virtual haptic object had to be slightly adjusted manually in order to achieve a sufficient alignment with the real haptic object. Providing a visual cue was essential to ensure that the participants hit the cavity with the hand-drill. Visual distraction and potential slight misalignment between the virtual and real haptic object caused by the manually adjusted alignment could have biased the perceived realism evaluation especially for Task 1 (correct position of the hand-drill for first contact with the cavity), and Task 3 (green highlighted virtual cavity to mark the maximum position). To control this, presence was measured. The presence ratings in both studies showed that the participants felt no or only little irritations in VR. Future studies should address the pure haptic perception. In four cases the software had to be restarted due to technical problems. Two subjects did the study without white noise. Some subjects required a break or took off the headsets for a second. In VR both haptic objects looked identical, but different from the real haptic object. The 3D model of the haptic object was oversimplified as there was no guiding mechanism for uniaxial movement visible. Although, no participant commented on this, it cannot be entirely ruled out, that a different behavior was subconsciously expected. However, as the visual and haptic behavior of the real and virtual haptic object were always consisted a potential bias would have affected both conditions. The different instructions when to stop pushing in Task 3 (blocking vs. highlighting), might have influenced the participants scoring. Despite raising stiffness to the technical maximum of the Virtouse 6D it was impossible to simulate an equal hard blocking for the virtual rendering condition as for the real haptic object. We assumed that many participants might not recognize this and keep on pushing, ultimately over pushing the Virtuose 6D. However, we instructed the participants to only include the pushing but not the blocking at the end into to their scoring. Nonetheless, a bias of the ratings cannot be ruled out. However, even if it had a negative effect it was the same for all haptic rendering methods in Task 3. We checked the data for a potential influence in comparison to Task 1 and Task 2. However, no obvious influence was visible. In case there was potential negative bias it would be the same for all haptic rendering methods in Task 3. For future studies the visualization of the real haptic object in VR should be highlighted green so that the visual feedback matches.

## Conclusion

Our findings suggest that for bimanual high force tasks a realistic haptic feedback can be achieved, although not with a single haptic rendering method and for worst-case scenarios like a steel/steel interaction. We could also show that the task plays an important role on the perceived realism. *Penalty* clearly emerged as the worst haptic rendering whilst there was no clear best. *Impulse* performed best for hard contact simulation. However, for simulating rotations and ‘pushing-in’-tasks with an increasing force *constraint* and *rigid body* delivered better results. Therefore, a combination of haptic rendering methods seems most promising. The methodical approach of performing an original study and confirming the results with a replication study significantly substantiate our conclusions, as only miniscule differences were found.

## Data Availability

The datasets generated during and/or analyzed during the current study are available from the corresponding author on reasonable request.
